# Book Reviews

**Published:** 1822-04

**Authors:** 


### Monthly Medical and Scientific Bibliography.

Vexat censura ...... Corvos.

### 1. GREAT BRITAIN.

Art. I.—*Two Voyages to New South Wales and Van-Diemen's Land. With a Description of the present Condition of that interesting Colony,* [of those interesting Colonies;] *including Facts and Observations relative to the State and Management of Convicts of both Sexes. Also Reflections on Seduction, and its general Consequences.* By Thomas Reid, &c. &c. 8vo. pp. 391. Longman and Co. London, 1822.

It will be asked by some of our readers, Why notice such a book as this in a medical Journal? Our answer is short. Mr. Thomas Reid is a surgeon in the Navy, who has passed his examinations at the Royal College of Surgeons with *eclat*, we believe, upon *every* question. He has twice served as surgeon and superintendent of both male and female convicts, shipped off for Botany Bay; and there is strong evidence, throughout his work, of his having acted the pious and praiseworthy office of missionary during the two voyages to that happy land; or, what amounts to the same thing, of having read impressive lessons on morals and religion to the unfortunate beings committed to his charge. In his work, moreover, he has entered into the consideration of the duties of a surgeon superintendent of a convict-ship, which, our readers will admit, cannot but form a very interesting subject of inquiry. Here, then, are many weighty reasons for announcing Mr. Thomas Reid's book in our Miscellany. We say, *announce* only; for, as to analyzing it, one might as well attempt to report or to analyze the speeches which the author, we understand, is occasionally in the habit of making at public meetings of charitable institutions.

The work contains some thoughts on transportation,—a journal of a voyage to Botany-Bay in the Neptune convict-ship,—the same of a second voyage in the Morley, with female convicts,—an account of the manner of disposing of convicts,—situation and duties of the surgeon superintendent,—and, lastly, general observations. In an Appendix, the author has favoured us with his reflections on seduction; in which we are at a loss whether to admire most the soundness of the principles, the perspicuity of the language, the felicity of the expressions, or the intimate knowledge the author has displayed of men and manners, particularly of foreign countries; in which, we have no doubt, Mr. Thomas Reid has resided long enough, and has mixed with society good enough, to authorize him to draw the conclusions wherewith he terminates his work on *transportation.*

---

Art. II.—*An Epitome of Pharmaceutical Chemistry: whereby the Art of Prescribing scientifically may be facilitated, and those Decompositions avoided, which, resulting from Combinations of incompatible Substances, often frustrate the Views of the Practitioner in their Medical Effects. Arranged according to the London Pharmacopœia.* By Rees Price, m.d. Member of the Royal College of Surgeons in London; Honorary Member of the Medical and Physical Society of Guy's Hospital, &c. &c. 12mo. pp. 59. Simpkin and Marshall, London, 1821.

This little work will be useful to many, and instructive to not a few. Notwithstanding the superior performance of Dr. Paris, and the extensive circulation its sound precepts must have acquired by this time, the art of prescribing is not much more advanced than it was previously to their appearance. We have happened to see prescriptions, at which a pharmaceutical chemist would have shuddered. We know an instance where the liquor ammoniæ acetatis was positively directed to be taken with magnesia and water, to the great annoyance of the poor patient, who found her mouth, not very gently, tickled by the caustic ammonia, which had naturally resulted from such a mixture. So various, and almost infinite, are the combinations effected by chemical laws, that the chances are greatly against the probability of our being able to take, indiscriminately, any two or three articles in the Dispensatory, which would not be found to destroy the identity of each other on being combined.

A knowledge of chemistry is as necessary to the prescribing physician, as minute anatomy is to the operative surgeon; and, in the absence of a complete knowledge of that science, all works, of the class of that which we are now noticing, are likely to be of service to the general practitioner, who has not had time to store his mind with much chemical information. Till such information has been acquired, he should regulate his practice conformably to the illustrations contained in the present little volume, which has the merit of being cheap, concise, and portable.

The book is drawn up in two columns: the left containing the articles of the London Pharmacopœia; the right indicating those substances with which each article is incompatible. A reference can thus be made to each remedial agent with the greatest promptitude, and a volume of information obtained by reading the opposite indications.

How many blunders such a plan will be the means of preventing, it is scarcely necessary for us to point out. At the same time, it should be observed, that we are by no means ultra-chemists; and that we are aware of the fact, that many old compounds have been successfully recommended, which defy every law of affinity and combination.

---

2. FRANCE.

ART. III.—*Journal de Physiologie Experimentale, &c.—Journal of Experimental and Pathological Physiology.* By F. MAGENDIE, Member of the Royal Institute. Vol. II. No. 1. 1822.

WE have recently received the first Number of this interesting periodical publication for the current year. It contains one or two important memoirs, by Dr. GASPARD and Dr. ROULLIN; a continuation of Mr. BELL's Anatomical and Physiological Researches into the Nervous System; Experiments on the Nervous System, by Mr. SHAW; and the Case of a Child, five years and seven months old, who was affected with Hydrophobia, in consequence of a Bite from a Mad Dog. The editor himself has supplied nothing of his own towards the compilation of the present Number. We trust that, having seated himself in the academical chair of the Institute, he will not relax—as some few have done, who work for the simple attainment of academical distinctions—in his efforts to promote the advancement of experimental physiology.

The memoir of Dr. Gaspard, on the Absorption of Purulent Fluids, is full of curious facts and useful information. The author details several experiments, made with a view to ascertain the effects of injecting pure pus into the veins of dogs, and comes to the following conclusions:—1. That a small quantity of pus may circulate through the blood-vessels, without producing death, provided it is again expelled from the system by a *critical* discharge of urine or fecal matter. 2. That, introduced, in small and repeated quantities, into the blood-vessels, it causes death, as it does when injected in a larger quantity. 3. That pus is susceptible of being absorbed; and that the greater number of symptoms which accompany hectic fevers seem to be due to the presence of pus in the animal economy.

In a second series of experiments, Dr. Gaspard injected vaccine matter into the veins of dogs, without producing any visible effect, either as to the appearance of any eruption, or with regard to any disturbance of the animal economy. Now, as VALENTIN and CHAUMONTEL succeeded in inoculating young dogs with vaccine matter, for the purpose of preserving them from the *distemper;* and ALIBERT, HUSSON, GEHIER, TEXIER, &c. did the same thing with regard to sheep; are we to suppose that vaccine virus is not communicable by the veins or through the circulation, but only through the medium of the skin?

There are other very important matters detailed in this curious paper of Dr. Gaspard, into which our limits forbid us to enter at present. We strongly recommend the perusal of it to our scientific readers.

---

### 3. DENMARK.

ART. IV.—*On the Use of Belladonna as a Preservative against Scarlet Fever.* (From the Danish Medical Journal, Bibliothek for Læger.)

THE observations made, twenty years ago, by HAHNEMANN, on the utility of belladonna in preserving from scarlet fever, have been confirmed by late experiments. In HUFELAND'S Journal for August, 1820, there is a statement of facts in favour of that medicine. Dr. BERENDT, (not Brandt, as some contemporaries have stated,) an Austrian physician, gave, morning and evening, a few drops of a solution of three grains of the extract of belladonna in one ounce of water, to 195 children exposed to the contagion of scarlatina; fourteen of whom, only, became infected with that disease, which they had in a very mild degree.

During the last epidemical scarlet fever which raged in Copenhagen and throughout Denmark, (1821,) this medicine proved extremely useful. Professor HERHOLDT, physician to the principal hospital in that metropolis, and one of its most eminent practitioners, has used a weaker solution of the extract, (two grains to one ounce of water,) and found it to answer the purpose of preserving several hundred children from the contagion. He observed, however, that the common and well-known effects of this medicine showed themselves in the little patients, when the dose of the solution, at any one time, amounted to twenty drops, and he therefore limited it to fifteen. The same author also prescribed it in a very recent epidemy; arrested, by it, the spreading of the contagion in families where the scarlet fever had made its appearance; and prevented its infecting most of the other families of which he was the attending physician.

Having remarked that the medicine ordered in the form of drops was more readily neglected in large families, Dr. Herholdt directed the extract of belladonna to be formed into lozenges, with a sufficient quantity of chocolate; and of these, each of which contained 1-32d part of a grain of the extract, he administered from one to three every morning and evening, according to the age of the patient. Out of nearly one hundred families, in which he had occasion to use the belladonna, one only was attacked by the scarlet-fever, during the epidemy, in spite of the remedy. It is not certain whether, in this family, the individuals had not been infected previously to their taking the belladonna, or whether they had neglected taking it regularly. Dr. Herholdt thinks that the medicine does not act as a preservative, unless it be given long before the person is exposed to the contagion.

---

### 4. AUSTRIA.

ART. V.—*Uebersicht der Vorfallenheiten in dem Entbindungt Institute, &c.—A Report of Cases that have occurred in the Military Lying-in Institution of the Medico-Chirurgical Josephine Academy at Vienna, between November* 1818, *and December* 1820.

DURING the two years, 129 soldiers' wives were admitted into the hospital, one, only, of whom had twins. Of the 130 children, 73 were males and 57 females. One hundred and eighteen presented the head; in one case, the head with one hand; footling, seven, (six of which presented one foot only, and the other both feet.) There were two nates, and two faces, presentations. Nine of the women were confined prematurely, and four miscarried. Of the children, 125 were born alive, and five still-born; six died after delivery,—viz. four boys and two girls; one of which was a six-months' child, which lived till the tenth day. The large proportion of premature labours is, very justly, attributed to the hard life which these women are obliged to lead.

All the labours terminated naturally. Among the tedious may be reckoned one in which the child weighed eleven pounds and measured twenty-two inches. In another case, the patient was five days in actual labour, although the child was only six months. The two cases of face-presentation were attended with considerable difficulty, but terminated without the use of instruments. Two of the women died; the one of typhous fever, the other of phthisis.

In endeavouring to account for some very acute pains in the head, which had supervened in one of the women after delivery, accompanied by defective secretion of milk and intermitting fever, the author of the report, W. I. SCHMITT, observes, that there existed a strong *polarity* between the uterus and the brain.

Icterus and ophthalmia neonatorum were the prevailing maladies among the infants. One of them is said to have died of suffocation, from a great accumulation of viscid mucus in the bronchia.

